# Splenic Abscesses in the Pediatric Population: A Literature Review of an Uncommon Entity

**DOI:** 10.7759/cureus.65607

**Published:** 2024-07-28

**Authors:** Nikhil Arora, Shefali Dhawan

**Affiliations:** 1 Pediatrics, Government Medical College, Patiala, Patiala, IND; 2 Radiodiagnosis, Government Medical College, Patiala, Patiala, IND

**Keywords:** splenic abscess, management of splenic abscess, diagnosis of splenic abscess, sign and symptoms of splenic abscess, causes of splenic abscess

## Abstract

Splenic abscess in the pediatric population is a rare but serious condition. Its incidence is reported to be between 0.05% and 0.7%. Splenic abscess is an infectious suppurative process with a discernible macroscopic filling defect in the subcapsular space or spleen parenchyma. Causes include bacterial infection through the hematogenous route or from locoregional spread such as the gastrointestinal tract, septic emboli, trauma, sickle cell anemia, and malarial infestation. Diagnosis is often delayed due to non-specific signs and symptoms. Symptoms include fever, abdominal pain, and palpable left upper abdominal swelling. Ultrasound and cross-sectional imaging such as CT scans are useful for the diagnosis of splenic abscess but contrast-enhanced CT scans are considered the gold standard because of their high sensitivity and specificity. Treatment options range from antimicrobial therapy, percutaneous drainage, and aspiration to surgical interventions such as splenectomy. As it has a high mortality rate, prompt diagnosis and appropriate treatment are essential for optimal outcomes.

## Introduction and background

Splenic abscess in the pediatric population is a rare but serious diagnosis. Although common in the adult population [1], it can be life-threatening in pediatric patients [2,3]. A splenic abscess is an infectious suppurative process with a discernible macroscopic filling defect in the subcapsular space or the spleen parenchyma [4]. Although it is commonly seen in immunocompromised children due to loss of the immunological and phagocytic function of the spleen, it can also be seen in immunocompetent children [5]. During the pre-antibiotic era, enteric fever resulting from *Salmonella enterica* serovar Typhi frequently caused splenic abscesses but its occurrence has decreased with the introduction of antimicrobial therapy [6]. Other causes of splenic abscess in the pediatric age group include bacterial infection through the hematogenous route or from locoregional spread such as the gastrointestinal tract, septic emboli, trauma, sickle cell anemia, and malarial infestation. Diagnosis is often delayed due to non-specific signs and symptoms [7]. Symptoms include fever, abdominal pain, and palpable left upper abdominal swelling [8,4]. Ultrasonography plays an important role in the diagnosis of splenic abscess, with a sensitivity of 90-98.8%. However, diagnosis needs to be confirmed by a contrast-enhanced CT scan which provides a sensitivity of 95-100% [2,3,9]. Treatment options range from splenectomy to conservative measures such as percutaneous aspiration or drainage along with antimicrobials [10,11]. Pediatricians need to consider these potential causes while evaluating a child with symptoms suggestive of a splenic abscess, as prompt diagnosis and appropriate treatment are essential for optimal outcomes.

## Review

Incidence

The most recent reported incidence of splenic abscess is between 0.05 and 0.7%. However, as imaging modalities are now being used more widely and effectively, the incidence has been increasing over time. There is no discernible shift in the gender predisposition, and the male-to-female ratio in children is roughly equal [3,12-14].

Causes

Splenic abscesses in children can occur due to various reasons, though they are relatively rare compared to adults. Most are caused by bacterial infections. These infections can arise from various sources such as the bloodstream/septicemia, adjacent organs, or through direct trauma. During the pre-antibiotic era, enteric fever resulting from *Salmonella enterica* serovar Typhi frequently caused splenic abscesses but its occurrence decreased with the introduction of antimicrobial therapy [6]. Infective endocarditis is also one of the common causes of splenic abscesses with *Streptococcus* and *Staphylococcus* being the most common bacterial pathogens [15]. *Salmonella* spp. is still thought to be the main cause of splenic abscesses, although the incidence of splenic abscesses caused by non-typhoidal *Salmonella* infection is rising rather than *Salmonella* serovar Typhi infection globally. *Salmonella* serovar Typhi and *Salmonella* serovar Paratyphi cause typhoid fever, whereas non-typhoidal *Salmonella* typically results in self-limiting acute gastroenteritis [16]. Even though non-typhoidal *Salmonella* can cause extraintestinal and invasive infections in individuals with compromised immune systems, the incidence is very low [16,17].

Another cause of splenic abscess is septic emboli. Abscess formation can result from bacteria entering the spleen through the bloodstream from distant locations, such as the heart or other infected organs. Embolization can occur to the normal spleen as in cases of septic endocarditis, intravenous drug abusers, or immunocompromised states. It can also occur in states of altered splenic architecture as in the case of sickle cell anemia, trauma, or vasculitis [18,19].

Additionally, a case report of a newborn who was hypothesized to have developed a splenic abscess as a result of umbilical venous catheter complication has been reported. This could have been caused by the infection from the catheter or by the catheter being positioned incorrectly, which could have traumatized the spleen [20].

Another cause is hematogenous spread. Bacteria can reach the spleen through the bloodstream, which can cause infection in the spleen. This may occur in patients with underlying conditions such as sickle cell anemia or those with immunocompromised states such as leukemia and human immunodeficiency virus/acquired immunodeficiency syndrome. It may also develop in cases of melioidosis, which is frequently associated with internal organ abscesses.

Splenic abscess formation and bacterial growth can be stimulated by traumatic injuries to the spleen. Although less frequent in children, accidents or physical trauma can end up causing this. Surgical procedures, such as splenectomy or splenic biopsy, may occasionally result in the development of splenic abscesses.

Although less frequent in developed regions, parasitic infections such as malaria can lead to splenic abscesses in children living in endemic regions. Structural alterations occur in the spleen during malarial infection ranging from simple enlargement to major consequences such as rupture, infarction, or hematoma [21,22]. A splenic abscess may occur after a splenic hematoma or infarction.

Rarely, splenic abscesses can also occur as a result of tuberculosis infection. Splenic abscess is one of the pathomorphological types of splenic tuberculosis. The other causes include miliary tuberculosis, nodular tuberculosis, calcific tuberculosis, and mixed-type tuberculosis [23].

Certain systemic illnesses, such as inflammatory bowel disease or endocarditis can also predispose children to develop splenic abscess [24]. In rare cases, fungal infections, tuberculosis, or infected cysts in the spleen can result in abscess formation.

All these possible causes should be kept in mind while evaluating a case of splenic abscess in a child for prompt diagnosis and appropriate treatment. Some of the reported cases of splenic abscess in this age group are presented in Table 1.

**Table 1:** A summary of previously published reports of children with splenic abscesses.

Author, year, and reference citation	Age (years)/Gender	Cause	Single or multiple abscesses/Size (largest; in cm)	Treatment and outcome
Jørgensen et al., 2022 [25]	15/Male	Salmonella enterica subsp. enterica	Single/17 cm	Ultrasound-guided percutaneous drainage and antibiotics, recovered
Salman et al., 2022 [26]	15/Male	Brucellosis	Multiple, <1 cm	Antibiotics, recovered
Sakulchit et al., 2021 [27]	8/Male	Melioidosis	Multiple	Antibiotics, recovered
Lee and Han, 2020 [28]	12/Male	Non-typhoidal Salmonella	Single/14.5 cm	Ultrasound-guided percutaneous drainage and antibiotics, recovered
Abiola et al., 2020 [29]	7/Female	Streptococcus sepsis	Single/12 cm	Partial splenectomy and antibiotics, recovered
Metlo et al., 2019 [30]	8/Female	Tuberculosis	Multiple/1.3 cm	Antibiotics, recovered
Ahmed et al., 2017 [31]	15/Male	Salmonella group B species	Single/15 cm	Partial splenectomy and antibiotics, recovered
Aslam et al., 2013 [20]	Preterm neonate/Female	Complication of umbilical venous catheter	Multiple/1.3 cm	Antibiotics and recovered
Yeom et al., 2011 [32]	14/Female	*Escherichia coli* sepsis	Multiple/4 cm	CT-guided percutaneous drainage and antibiotics, recovered
Sithasanan et al., 2007 [33]	4/Female	Acute lymphoblastic leukemia and fungal sepsis	Multiple	Splenectomy and antibiotics, recovered

Signs and symptoms

Splenic abscess in a child can present with various non-specific signs and symptoms, hence, diagnosis is often delayed in these cases [7]. Fever is the most common symptom occurring in 71-88% of the cases [3]. The child may complain of pain in the left upper abdomen, which can range from mild to severe. The left upper quadrant of the abdomen may be tender. Patients may also present with Kehr’s sign due to referred pain to the ipsilateral clavicular region as a result of abscess-induced irritation of the phrenic nerve [34]. Other symptoms may include nausea, vomiting, fatigue, weight loss, and splenomegaly. In severe cases, the infection can spread rapidly, and the child may develop symptoms of septic shock, including tachycardia, hypotension, and altered mental status [35]. In cases of pyrexia of unknown origin, diagnosis of splenic abscess can be suspected [12].

It is key to remain cognizant that symptoms might change based on the child’s age, the underlying etiology, and the presence of complications. If a splenic abscess is suspected, prompt medical attention is necessary for diagnosis and treatment.

Investigations

Certain blood investigations are crucial for diagnosis and to guide treatment in cases of suspected splenic abscesses. In cases of infection, the white blood cell (WBC) count, specifically the neutrophil count, is often elevated. A triad of fever, pain in the left quadrant of the abdomen, and an increase in the WBC count is suggestive of splenic abscess, as seen in most cases [3,36]. However, leukocytosis may not be seen in cases of splenic abscess in immunocompromised children.

Markers of inflammation such as C-reactive protein and erythrocyte sedimentation rate may be elevated. Liver function tests may be deranged if the liver is involved or in cases of sepsis. Renal functions can also be altered. Blood cultures help guide antimicrobial therapy.

These blood tests aid in the diagnosis of splenic abscess, infection severity assessment, antibiotic medication guidance, and treatment response monitoring. Treatment planning and diagnosis may also require further imaging, such as an ultrasound or cross-sectional studies depending on the clinical presentation and severity of the disease.

Depending on the imaging modality used and the age of the patient, the appearance of the spleen may vary. Any type of heterogeneous echotexture of the spleen on an ultrasound scan should be a cause for concern. On ultrasound, a bacterial abscess is seen as a large, cystic lesion with a defined periphery and no central vascularity on color Doppler [37]. Intralesional gas is also a pathognomic of pyogenic abscesses. The components of fungal splenic abscesses are necrotic tissue, purulent debris, and fungus, enclosed by layers of persistent inflammatory cells and surrounding fibrosis. This results in the “wheel-within-a-wheel,” “bull eye,” or “target” appearance of fungal microabscesses on the ultrasound. This consists of a peripheral hypoechoic fibrotic zone, an enclosed inflammatory hyperechoic zone portion, and a hyperechoic center with purulent material, necrotic debris, and fungal elements. Ultrasonography can also be performed bedside for the detection of splenic abscesses in sick patients for early diagnosis and treatment.

Ultrasound and CT scans are useful for diagnosing splenic abscesses, but CT scan is considered the gold standard because of its high sensitivity and specificity [14]. It provides detailed cross-sectional images of the spleen and surrounding structures. It also helps determine the size, location, and characteristics of the abscess, which are important factors in deciding the most appropriate treatment plan. On a CT scan, a well-defined lesion with a center of low attenuation (ranging from 20 to 40 HU) is seen, depending on the amount of proteinaceous material [37]. It can also show intra-abscess gas formation. CT and ultrasound, however, are not sensitive enough to distinguish between an infected, non-parasitic, splenic cyst or pseudocyst.

Another valuable tool in the evaluation of splenic abscesses is MRI, but it is not considered the first-choice imaging modality. MRI provides good soft tissue resolution and can help differentiate between different types of tissues, which is useful in evaluating the extent of the abscess and its surrounding tissue. MRI shows areas of fluid signal intensity, i.e., low signal intensity on T1-weighted sequences and high signal intensity on T2-weighted sequences [37]. Peripheral and perilesional enhancement on post-contrast T1-weighted fat-saturation imaging is also seen.

An X-ray of the abdomen often shows non-specific findings such as gas collection in the upper left quadrant [29]. It can also show an area of increased density which suggests splenomegaly. An X-ray of the chest can show elevation of the left hemidiaphragm and sometimes pleural effusion [36].

Confirmation of diagnosis is made by aspiration, followed by microscopic examination and cultures of the abscess. The most common organisms cultured include staphylococci, streptococci, *Salmonella*, and *Escherichia coli* [2]. Bacterial infection is more commonly seen, whereas fungal abscess is usually seen in immunocompromised children [38]. Cultures also guide about the antimicrobials to be used for treatment.

Treatment

Antibiotic Therapy

The treatment of splenic abscess involves a combination of antibiotics and, in some cases, drainage procedures. Antimicrobial therapy is the cornerstone of treatment for splenic abscesses. Empirical antibiotics are often initially started until culture and sensitivity results are available. The optimal duration of antibiotic therapy remains uncertain; however, treatment periods lasting for six to nine weeks have been reported [18,39]. The duration of the course of antibiotics depends on the severity of the infection and the response to treatment.

Percutaneous Drainage and Aspiration

Traditionally, splenectomy was recommended [15] for splenic abscesses but now percutaneous drainage is preferred [10]. This involves using imaging guidance, such as ultrasound or CT scan, to place a needle or catheter directly into the abscess cavity to drain the pus and fluid. Percutaneous drainage is done along with antibiotic therapy. This procedure can help relieve symptoms much faster, facilitate resolution of the abscess, and may reduce the duration of antibiotic therapy. Although a splenic abscess with a diameter of 10 cm or less is treated with percutaneous aspiration and drainage procedure, in a recent case report of a 12-year-old child, a splenic abscess with a diameter of 14.5 cm was treated with percutaneous drainage and antibiotics [10,28]. Usually, percutaneous drainage is done in cases of single splenic abscess. However, in the past few years, it has been done successfully in cases of multiple abscesses [12,36]. Complications of percutaneous drainage include hemorrhage, risk of injury to surrounding structures, fistula formation, risk of spread of infection, pneumothorax, pleural empyema, and minor complications such as local pain and subcapsular hematoma. In complex cases of poorly accessible splenic abscess or when the abscess is near any major vessel, CT-guided drainage and aspiration can be done. Hence, percutaneous drainage is a well-accepted, less invasive, and effective procedure to be done in cases where the goal is to preserve the spleen.

Supportive Therapy

Along with this, supportive care needs to be done. Intravenous fluids may be administered to maintain hydration, and analgesics may be given for pain relief. Monitoring of the patient’s clinical status, its response to treatment, and radioimaging such as ultrasound or CT scan are essential to assess the resolution of the abscess and detect any complications.

Surgical Interventions

In some cases when there are complications such as abscess rupture or when percutaneous drainage is not possible or ineffective, surgical intervention might be required. Surgical alternatives encompass open drainage or, in certain situations, splenectomy. Splenectomy is generally regarded as a final option due to the potential for post-splenectomy complications, which may lead to an increased vulnerability to specific infections. Therefore, total splenectomy is avoided in children. Earlier total splenectomy was considered standard surgical treatment, but now spleen-preserving techniques such as partial septectomy or percutaneous drainage are the preferred options due to the important immunological and phagocytic function of the spleen [40]. The choice of treatment depends on the anatomical location of the spleen, extent of infection, etiology, and viability of the spleen. A 15-year-old with a large splenic abscess was treated with partial splenectomy, as the abscess was in the lower pole of the spleen and the remaining spleen appeared healthy [31]. In patients with multifocal abscesses and in immunocompromised states, total splenectomy is preferred to prevent recurrence and infections [32].

Overall, the treatment approach for splenic abscesses should be individualized based on factors such as the size and site of the abscess, the patient’s clinical condition, and the expertise available for its management. Untreated splenic abscesses have high mortality as they may spread to other organs. Prompt initiation of appropriate antimicrobial therapy with timely consideration for drainage procedures when indicated are key principles in the management of splenic abscess.

## Conclusions

Splenic abscess is a rare entity, occurring more commonly in immunocompromised hosts. Diagnosis of splenic abscess is often delayed due to the non-specific signs and symptoms. Ultrasound and CT scans are useful for diagnosing splenic abscesses, but contrast-enhanced CT scans are considered the gold standard. Traditionally, splenectomy was done in cases of a splenic abscess, but, nowadays, spleen-preserving techniques such as percutaneous drainage and aspiration along with antibiotics are preferred options. Pediatricians need to consider potential causes while evaluating a child with symptoms suggestive of a splenic abscess, as prompt diagnosis and appropriate treatment are essential for optimal outcomes.
